# Editorial: Experimental and computational approaches in meniscus mechanics and mechanobiology

**DOI:** 10.3389/fbioe.2024.1479315

**Published:** 2024-09-02

**Authors:** Francesco Travascio, Andreas M. Seitz, Petri Tanska

**Affiliations:** ^1^ Department of Mechanical and Aerospace Engineering, University of Miami, Coral Gables, FL, United States; ^2^ Department of Orthopedic Surgery, University of Miami, Coral Gables, FL, United States; ^3^ Max Biedermann Institute for Biomechanics at Mount Sinai Medical Center, Miami Beach, FL, United States; ^4^ Institute of Orthopaedic Research and Biomechanics, Ulm University Medical Centre, Ulm, Germany; ^5^ Department of Technical Physics, University of Eastern Finland, Kuopio, Finland

**Keywords:** meniscus biomechanics, mechanobiology, artificial intelligence, knee simulator, meniscal tears, meniscal degeneration

## The complex biomechanics of knee menisci

The knee menisci are remarkable fibrocartilaginous structures that play a crucial role in maintaining joint congruency, facilitating load transmission, and providing load dampening. These semilunar wedges, situated between the femur and tibia, enable a congruent sliding motion, ensuring smooth and efficient joint mechanics (Sanchez-Adams and Athanasiou, 2009). Their unique shape and composition endow them with heterogeneous, anisotropic, and nonlinear mechanical properties, allowing them to withstand loads that can be multiple times the individual’s body weight (Walker et al., 2015). The menisci are composed of a complex network of collagen fibers, proteoglycans, and water, which together form a tissue capable of withstanding significant mechanical stress. The unique arrangement of collagen fibers contributes to the anisotropic mechanical properties of the meniscus (De Rosa et al., 2022; Morejon et al.), enabling it to absorb and distribute loads effectively. This structural integrity is crucial for the proper functioning of the knee joint, as it ensures even distribution of weight and reduces the risk of injury to the articular cartilage. However, despite their robust nature, meniscal injuries are alarmingly common, particularly among the active population (Baker et al., 1985). Moreover, in elder population, degenerative processes of the meniscus, potentially leading to osteoarthritis, are common (Fox et al., 2015).

## Research challenges and future directions

Understanding the complexities of meniscal biomechanics and mechanobiology is vital for developing effective treatments and interventions. To date, several research challenges need to be addressed to optimize modeling strategies for studying meniscal biomechanics. For instance, the evaluation of load distribution in the tissue, especially in relation to different occupational activities, is crucial for developing targeted interventions. Current pertinent efforts include the use of multiple imaging modalities and sophisticated analysis techniques (both *in-vivo* and *ex-vivo*) (Schwer et al., 2020; Peloquin et al., 2023). Severyns et al. conducted a feasibility study in which they combined magnetic resonance imaging and digital volume correlation in clinical 7T MRI to study meniscus biomechanics. Also, the investigation of the effects of surgical and pharmacological treatments on animal models can provide insights into potential human applications. However, preclinical animal models are not currently performed in a standardized fashion. Hence, the development of “Best Practice” standards would increase reproducibility of experiments, thus accelerating advancements in translational research (Bansal et al., 2017). Furthermore, developing experimental and/or computational methods to evaluate the mechanical interactions between the extracellular matrix components (e.g., collagen fibers) and meniscus cells in different regions of the meniscus and under complex physiological loading conditions can enhance our understanding of tissue mechanics and its mechanisms of injury (Danso et al., 2015; Simkheada et al., 2023; Jeong et al., 2024; Andress et al., 2022). Experimental and computational models could also be adopted to predict disease progression and evaluate the efficacy of pharmacological or surgical interventions (Travascio and Jackson, 2017). Bartolo et al. presented a proof-of-concept ovine knee simulator that could be utilized to impose *in-vivo* gait loading for, e.g., meniscus replacement tissues. These kinds of approaches could enable the development of different surgical strategies without the need for live animals. In the future, balancing the use of generalized models for population analyses with subject-specific models based on detailed medical imaging will improve the personalization of treatments. In pursuing this effort, we anticipate an increasing use of artificial intelligence-based models into diagnostic, preventive, and treatment strategies, which will revolutionize meniscal care by providing more accurate and timely insights (Zhao et al., 2024).

## Final considerations

The menisci of the knee are critical to joint function, and understanding their biomechanics and mechanobiology is essential for advancing medical treatments. The interplay between mechanical properties, tissue composition, and cellular dynamics presents a complex challenge for researchers. However, with continued advancements in computational modeling, experimental methods, and validation techniques, we can develop more effective and personalized interventions for meniscal injuries and degeneration. For instance, in this Research Topic, it will be shown how an ovine knee simulator may be able to mimic physiologically relevant *in vivo* kinematics and contact pressures (Bartolo et al.), thus suggesting that this tool can be used for refining and de-risking new meniscal implants and surgical procedures. It is also shown how the development of new imaging approaches based on MRI-based deep learning (Ying et al.) digital image correlation (Severyns et al.) may enhance our accuracy in diagnosing tears to the meniscus, and help us understanding how these injuries affect meniscal mobility and extrusion, which may lead to the development of osteoarthritis, if not treated.

Hence, the issues addressed in this Research Topic open the door to new challenges, such as the refinement of knee simulators to evaluate a broader range of meniscal implants and surgical techniques. This could include testing different materials, fixation methods, and evaluating the long-term durability of implants. Another major foreseeable challenge will be the integration of AI systems into clinical workflows to enhance early detection and treatment planning for meniscal tears, as well as the use of weight-bearing MRI systems to hopefully provide more personalized treatment strategies. Furthermore, given the mechanical differences observed between healthy and osteoarthritic menisci (Weiske et al.), research should explore targeted therapies aimed at restoring or preserving meniscal function in osteoarthritic patients. This could involve the development of new biomaterials or drug delivery systems that mitigate degeneration.
